# Multimodal evaluation of the effects of low-intensity ultrasound on cerebral blood flow after traumatic brain injury in mice

**DOI:** 10.1186/s12868-024-00849-0

**Published:** 2024-02-13

**Authors:** Huiling Yi, Shuo Wu, Xiaohan Wang, Lanxiang Liu, Wenzhu Wang, Yan Yu, Zihan Li, Yinglan Jin, Jian Liu, Tao Zheng, Dan Du

**Affiliations:** 1 First Hospital of Qinhuangdao, No.258, Culture Road, Seaport District, Qinhuangdao, Hebei Province China; 2 Graduate School, Chengde Medical University, Chengde, Hebei Province China; 3 Beijing Key Laboratory of Neural Injury and Rehabilitation, China Rehabilitation Research Center, Beijing, China; 4 Peking University, Beijing, China; 5 Northeastern University at Qinhuangdao of Information Science and Engineering, Qinhuangdao, Hebei Province China

**Keywords:** Traumatic brain injury, Transcranial focused ultrasound stimulation, Angiogenesis Laser speckle imaging, Vascular endothelial growth factor

## Abstract

Traumatic brain injury (TBI) is one of the leading causes of death and disability worldwide, and destruction of the cerebrovascular system is a major factor in the cascade of secondary injuries caused by TBI. Laser speckle imaging (LSCI)has high sensitivity in detecting cerebral blood flow. LSCI can visually show that transcranial focused ultrasound stimulation (tFUS) treatment stimulates angiogenesis and increases blood flow. To study the effect of tFUS on promoting angiogenesis in Controlled Cortical impact (CCI) model. tFUS was administered daily for 10 min and for 14 consecutive days after TBI. Cerebral blood flow was measured by LSCI at 1, 3, 7 and 14 days after trauma. Functional outcomes were assessed using LSCI and neurological severity score (NSS). After the last test, Nissl staining and vascular endothelial growth factor (VEGF) were used to assess neuropathology. TBI can cause the destruction of cerebrovascular system. Blood flow was significantly increased in TBI treated with tFUS. LSCI, behavioral and histological findings suggest that tFUS treatment can promote angiogenesis after TBI.

## Introduction

Traumatic brain injury (TBI) is currently the leading cause of death and long-term disability, accounting for 30% of all injury-related deaths, globally [1, 2]. In China, the annual incidence of craniocerebral trauma is 55–64/100,000, resulting in nearly 100,000 deaths and hundreds of thousands of disabilities every year, which is a serious public health problem [3]. A study found that 90% of the patients who died from TBIs had ischemic injuries [4]. Furthermore, cerebral ischemia is one of the most important causes of secondary brain injury after TBI [5]. Secondary brain injury caused by TBI is caused mainly by an abnormal local vascular system with the destruction of the cerebrovascular system being the main factor leading to a series of secondary injury cascade reactions caused by TBI [6].

As early as 20 years ago, the potential of using ultrasound as a new tool to regulate angiogenesis began to emerge [7–10], leading to the treatment of vascular system abnormalities by controlling angiogenesis becoming an important direction in neuroscience research. Therapeutic ultrasound is an essential therapeutic method in physical therapy, especially tFUS, which has attracted extensive attention in the field of neuroscience because of its high spatial resolution, high penetration depth, and noninvasive properties. Previous studies have shown that tFUS can promote bone healing effectively by enhancing and regulating the expression of the vascular endothelial growth factor (VEGF) during early fracture healing and subsequent chondrogenesis [11, 12]. The tFUS can also induce angiogenesis during myocardial ischemia [13]. Another study also found that tFUS intervention can stimulate the production of a variety of cytokines, specifically regulating blood vessel generation and axonal growth [14, 15] Moreover, a recent study found that tFUS significantly improved cognitive dysfunction in mice with vascular dementia by increasing the cerebral blood flow (CBF) in the hippocampus [16].

Based on the neurovascular coupling mechanism,functional near-infrared spectroscopy (fNIRS) is a kind of brain functional imaging technique, But the disadvantage is that the collection needs to be repeated many times, and too long experiment time will lead to low frequency oscillation.Arterial spin labeling (ASL) is a non-invasive, non-radiative tissue perfusion imaging method without additional tracer injection. The main disadvantages of ASL are the challenges of producing images with low signal-to-noise ratio (SNR), uneven magnetic fields, shortened T2* values, SAR limitations, etc.Different from fNIRS and MR-basedASL, Laser speckle imaging (LSCI) is a type of wide field, non-scanning imaging technique, with high spatial and temporal resolutions for the long-term, dynamic assessment of the hemodynamic response to related neuronal activity.

Laser speckle imaging (LSCI) is a type of wide field, non-scanning imaging technique, with high spatial and temporal resolutions for the long-term, dynamic assessment of the hemodynamic response to related neuronal activity; the quantitative analysis of CBF has been used widely in animal studies, clinical diagnoses, and intraoperative monitoring [17–21].

In this study, we evaluated the effects of tFUS on motor function in TBI mouse models by assessing the improvement of the degree of nerve injury scores. We also monitored the changes in CBF in the injured cortex of TBI mice using LSCI and observed the damage to the neurons in the injured cortex and their recovery after treatment using Nissl staining.

## Materials and methods

### Animals and experimental groups

Forty-five healthy C57BL/6 mice (All males, weighing 20–22 g, Beijing Weitong Lihua Experimental Animal Company.) were used in this study. All procedures were performed under institutional review and approved by the Medical Ethics Committee of Qinhuangdao Municipal No.1 Hospital. Mice were randomly divided into three groups: the Sham group(*n* = 6), the TBI group(*n* = 16), and the TBI + tFUS group(*n* = 16). Seven mice were excluded from the study, including four that had no neurological defects and three that died during the experiment.

Sham group: No mechanical striking was performed after bone window opening.

TBI group: After the controlled cortical impact (CCI), sham tFUS was administered for 10 min every day.

TBI + tFUS group: After the CCI procedure, tFUS was administered for 10 min every day.

### Establishment of controlled cortical impact (CCI) model

The mice were fed separately for three days in advance and then fasted for 8 h before surgery. The mice were anesthetized using isoflurane inhalation, and the anesthetized mice were fixed in a prone position using a stereotaxic device. The skin was disinfected routinely, and the periosteum was dissected in the middle to expose the left parietal bone. The skull was drilled 2 mm behind the anterior skull and 2 mm on the left side of the midline to expose the parietal lobe with a 5 mm diameter cranial head to maintain the integrity of the dura. A controlled cortical impact was generated using a cranial precision striker (68,099, RWD, Shenzhen, China) were used to strike at a speed of 3.5 m/s [22], a depth of 1 mm and a retention time of 1 s to simulate moderate brain injury in the left parietal lobe. After complete hemostasis, bone wax was used to close the bone window, and the scalp was sutured. In the sham group, the craniotomy window was closed using bone wax without any blows, while the other processes were the same as those in the TBI + tFUS group. After the operation, the mice were fed alone and attention was paid to their heat preservation. All the mice were fully awake approximately 2–3 h after the surgery. All the mice in the TBI + tFUS and TBI groups showed a loss of hind limb movement ability on the injured side, unilateral hemiplegia (on the injured side), decreased muscle tension below the injured plane, no response to acupuncture on the injured side, and an inability to walk in a straight line. This shows that the model was established successfully.

### The tFUS protocol

The ultrasonic stimulation system and parameters are the same as our previous studies [23]. In the tFUS system, two connected function generators (AFG3022C; Tektronix, USA) were used to generate the pulsed signals. The pulsed signal from the second generator was amplified using a linear power amplifier (E&I240 L; ENI Inc., USA) and transmitted to an unfocused ultrasound transducer (V301-SU; Olympus, Japan). The mice were anesthetized using isoflurane inhalation. The ultrasound transducer was applied to the target region in the injured cortical areas using a conical collimator. The total stimulation duration was 10 min.The ultrasound fundamental frequency (FF) and pulsed repetition frequency (PRF) were 500 kHz and 1 kHz, respectively. The ultrasound stimulation duration (SD) and tone-burst duration (TBD) were 400 ms and 0.5 ms, respectively [24]. The ultrasound pressure was measured by a calibrated needle-type hydrophone (HNR500; Onda,USA), and the spatial peak average intensity (Isppa) was 2.6 W/cm2.(Table 1).

**Table 1:** Ultrasound parameters

FF	PRF	SD	TBD	Isppa
500 kHz	1 kHz	400 ms	0.5 ms	2.6 W/cm^2^

### Neurological severity score (NSS)

In this study, the neurological function of the mice was evaluated using the NSS (Table 2). The overall neurological function of the mice was evaluated at 1, 3, 7, and 14 days after the operation. The results were repeated three times, and the average value was calculated as the result of the day.

**Table 2:** Neurological severity scores of Mice

Task	Description	Points (success/failure)
Exit circle	Ability and initiative to exit a circle of 30 cm diameter within 3 min	0/1
Mono/hemiparesis	Paresis of upper and/or lower limb of the contralateral side	0/1
Straight walk	Alterness,initiative and motor abilify to walk straight	0/1
Startle reflex	Innate reflex (bounce in response to a loud hand clap)	0/1
Seeking behavior	Physiological behavior as a sign of “interest” in the environment	0/1
Beam balancing	Ability to balance on a beam of 7 mm width for at least 10s	0/1
Round stick balancing	Ability to balance on a round stick of 5 mm diameter for at least 10s	0/1
Beam walk:3 cm	Ability to cross a 30-cm long beam of 3 cm width	0/1
Beam walk:2 cm	Same task but with increased difficulty on a 2-cm wide beam	0/1
Beam walk:1 cm	Sanie task but with increased difficulty on a 1-cm wide beam	0/1
Maximal 5 core		10

### Laser speckle imaging (LSCI)

**Fig. 1:**
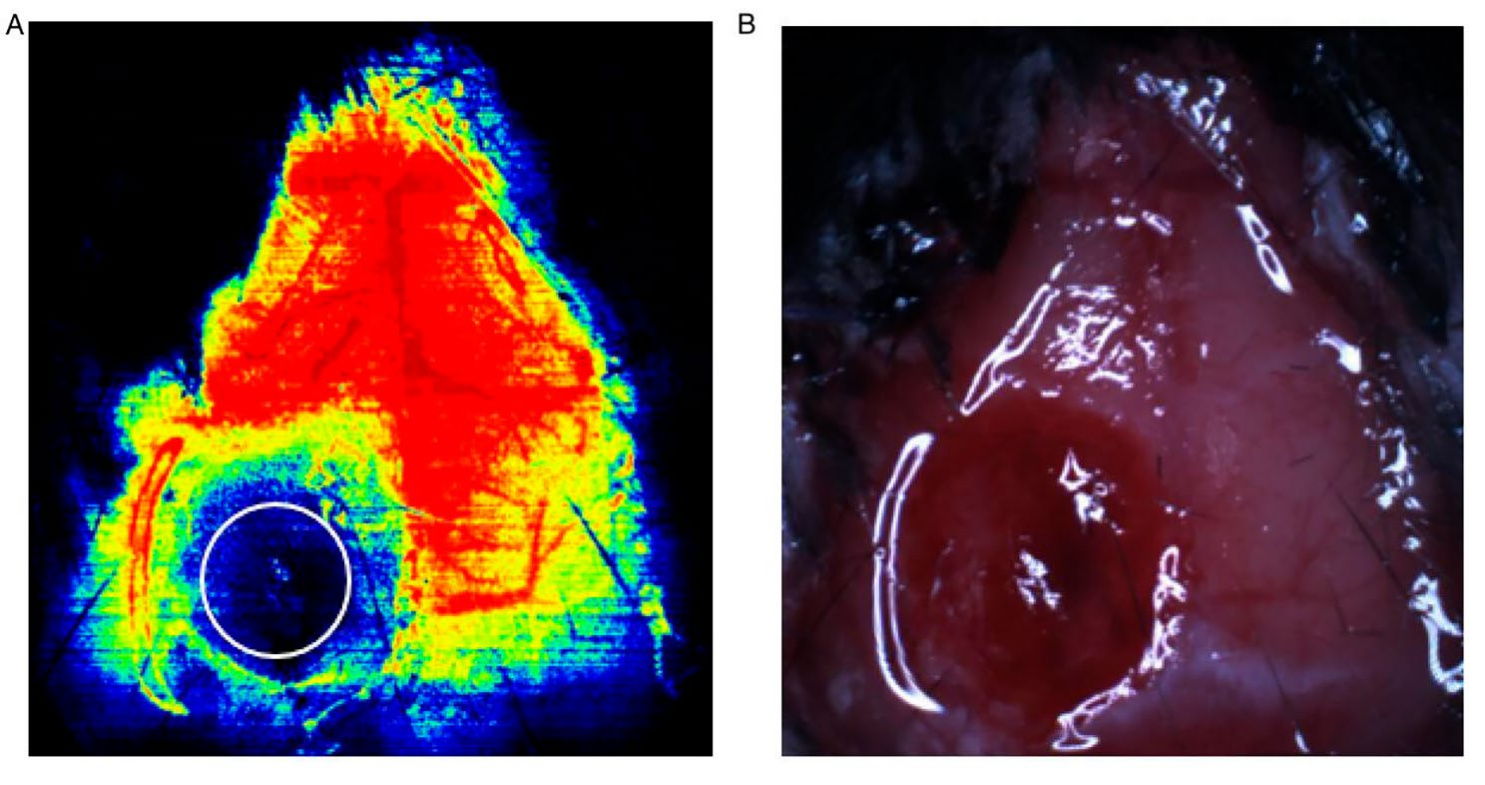
(**A**) In laser speckle imaging images, the white circle is the area of trauma. (**B**) In living brain tissue, the damaged area is bleeding and swollen, corresponding to the white circle

During the induction of anesthesia using isoflurane inhalation, the heads of the mice were fixed in the stereotactic frame; the head hair was cut off; a midline incision was made in the skin and subcutaneous tissue in the temporal side of the head; and the frontal, parietal, and occipital surface residual subcutaneous tissue was removed as much as possible before exposure of the anterior fontanelle bilateral parietal lobes. Using the RWD LCSI system, the relative position of the machine and the animal was adjusted so that the red cross point of the indicator laser was located in the center of the brain. After auto-focusing, the perfusion image was collected using high-resolution LSCI. CBF in the ROI was calculated after 10 s of monitoring. The size of the ROI was 85 × 85(Fig. 1).

### Pathology assessment

#### Nissl staining

After neurobehavioral tests, mice were euthanized by cardiac perfusion. The mice were anesthetized with isoflurane and perfused via the heart with cold PBS (pH 7.4), followed by 4% paraformaldehyde in 0.1 M PBS. The fixed brain tissue was embedded in paraffin and sectioned at a thickness of 0.5 cm. The paraffin sections were dewaxed with xylene and soaked in a gradient of ethanol. The sections were stained in solution A (Cresyl violet stain) at 56 °C for 60 min and rinsed with phosphate-buffered saline (PBS). The cells were placed in solution B (Nissl Differentiation) for several seconds to 2 min until the background color was nearly colorless, and the morphology of the neurons and Nissl corpuscles was observed under a microscope.

#### VEGF

VEGF is expressed mainly in the cell cytoplasm and appears as a brownish-yellow colored substance. The samples were sectioned 5 μm; the slices were placed on slides; the laboratory oven set was at 60℃ for 2 h; the slices were dewaxed and hydrated with ethanol, soaked in PBS for 10 min and in citrate buffer for antigen repair, washed twice with PBS, inactivated with 3% H2O2 for 30 min, and finally washed twice with PBS. The goat serum was sealed for 2 h, and a primary VEGF antibody and secondary antibody (kit provided by) were used. The goat serum was refrigerated overnight at 4 ℃ and washed twice with PBS. A universal antibody IgG antibody was used as the secondary antibody. The goat serum was incubated in a temperature box for 30 min, washed twice with PBS, and stained twice with 3,3’-diaminobenzidine buffers. The samples were re-stained with hematoxylin, dehydrated with ethanol, dried naturally, sealed, and examined under a biological microscope.

### Statistical analysis

The measurement data were expressed as means ± standard deviations. The data were analyzed statistically using the GraphPad Prism standard software package (version 7.00, La Jolla, CA, USA). The Shapiro-Wilk test was used to confirm the normal distribution of the data. The Levene test was used to confirm the homogeneity of variance of the data. The intra-group correlation coefficients of two repeated measurements were calculated. For neurobehavioral scores and CBF, each group was compared using a repeated two-factor analysis of variance (ANOVA), followed by the Tukey’s test. Mauchly’s test of Sphericity is used to test whether the variances of the differences between different measurements are equal. If sphericity is not satisfied, Greenhouse-Geisser method is adopted for correction. For VEGF, each group was compared using one-way ANOVA. Statistical significance was set at *P* < 0.05.

## Results

### Behavioral scores

**Fig. 2:**
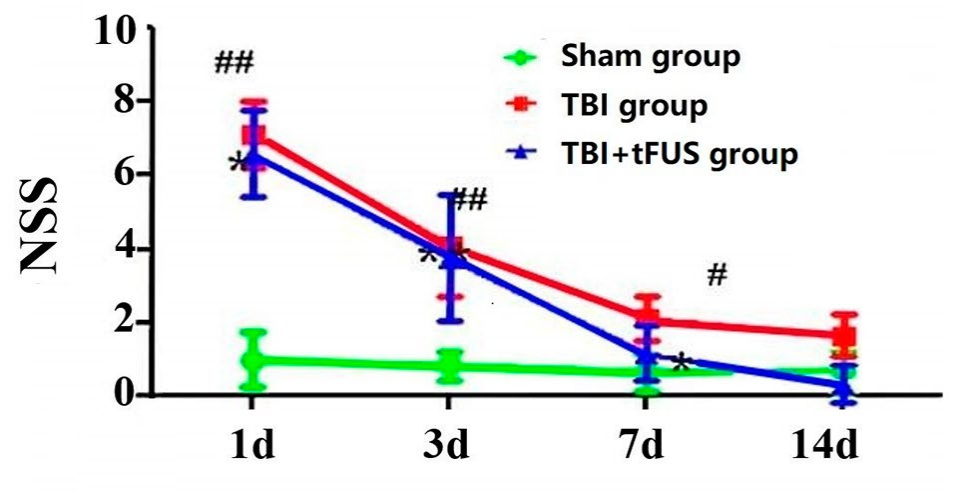
The Neurological Severity Score (NSS) of rats in the sham, TBI and TBI + tFUS groups at 1, 3, 7 and 14 days after TBI. Data are presented as mean ± SD. ##*P* < 0.01, Sham vs. TBI. #*P* < 0.05, Sham vs. TBI. **P < 0.01, TBI vs. TBI + tFUS. *P < 0.05, TBI vs. TBI + tFUS.

The results showed that, compared with the NSS scores at 3,7 and 14 days, the NSS score at 1 days was the highest (*p* < 0.001), and the NSS scores at 3, 7 and 14 days continued to decline (Fig. 2). Compared with Sham and TBI + tFUS groups, the NSS score of TBI group was the highest, and the NSS score of TBI + tFUS group was lower than that of TBI group.

### Cerebral blood flow

**Fig. 3:**
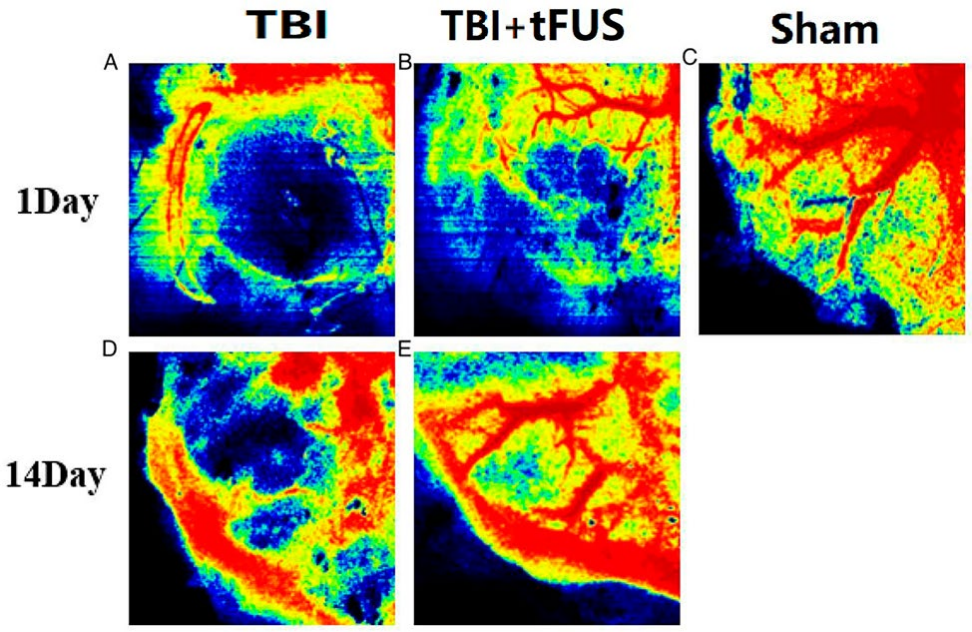
(**A-C**) Vascular conditions in the TBI, TBI + tFUS and Sham groups at day 1. (**D, E**) Vascular conditions of the TBI and TBI + tFUS groups at day 14

**Fig. 4:**
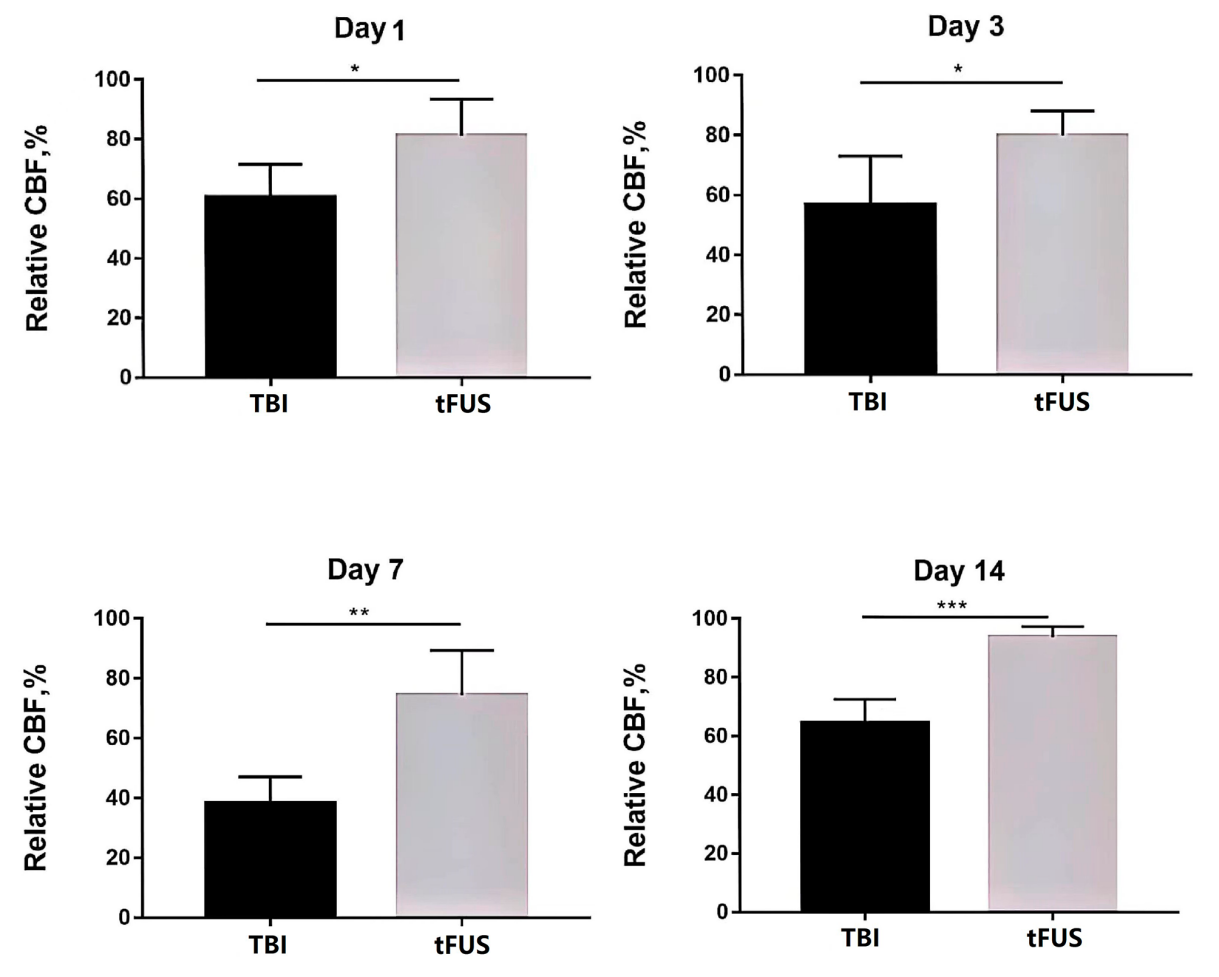
rCBF values at 1, 3, 7 and 14 days after TBI in each group, rCBF values = affected side CBF/ healthy side CBF*100%, Two-way ANOVA for repeated measures, followed by Tukey’s post hoc test. *P < 0.05, **P < 0.01, and ***P < 0.001

Relative cerebral blood flow (rCBF), as measured by Laser speckle imaging (Fig. 3), was evaluated in TBI and tFUS + TBI mice. On the 1st and 3rd day after surgery, rCBF decreased in both the TBI group and the tFUS + TBI group, and there was a statistical difference in rCBF between the TBI group and the tFUS + TBI group (*P* = 0.024, *P* = 0.021, *P* < 0.05). On the 7th day after surgery, the rCBF of the TBI group and tFUS + TBI group decreased to the lowest level, and the rCBF of the TBI group and tFUS + TBI group was statistically different (*P* = 0.002, *P* < 0.01), on the 14th day after surgery, the rCBF of the TBI group and tFUS + TBI group increased, and the rCBF of the TBI group and tFUS + TBI group was statistically different (*P* < 0.001) (Fig. 4).

### Nissl’s staining

**Fig. 5:**
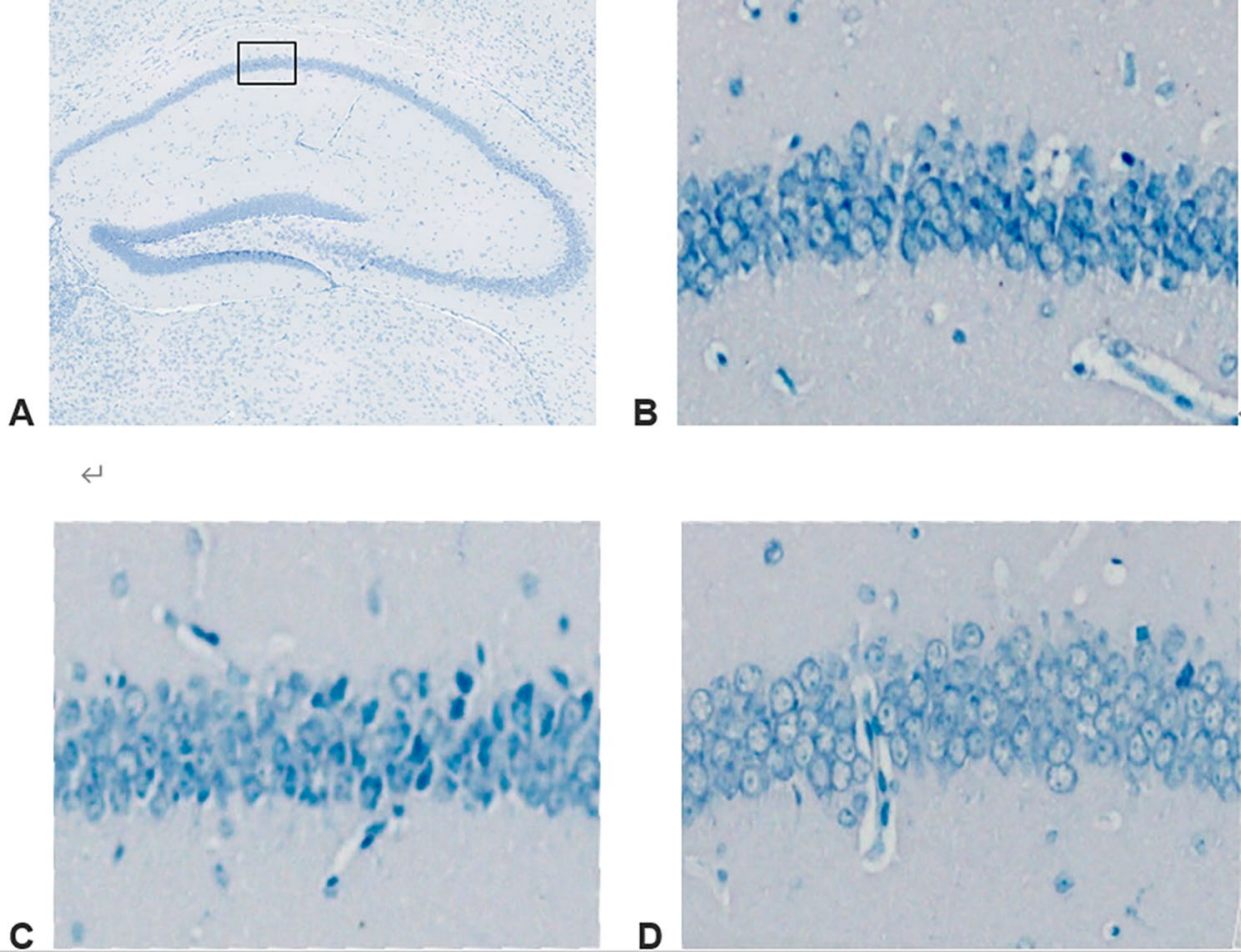
**A**. Nissl staining in the hippocampal CA1 area of the Sham, TBI and TBI + tFUS groups. **B**. the Sham group. **C**. the TBI + tFUS group. **D**. the TBI group

**Fig. 6:**
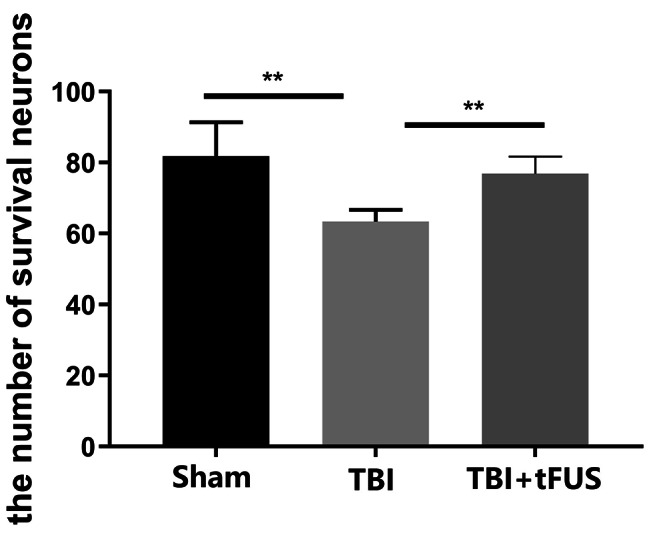
Survival cell counts in the hippocampal CA1 area of the Sham, TBI and TBI + tFUS groups. **P < 0.01

In the sham group, the neurons in the hippocampal CA1 area were large and round, the cytoplasm was evenly colored, and the Nissl bodies around the cytoplasm were uniformly blue with intact and normal shape. In the TBI and tFUS + TBI groups, the neurons in the hippocampal CA1 area were pyknotic, forming dense plaques; the gaps between cells became larger; the cell arrangement was loose; and the number of regular morphological neurons was small (Fig. 5). Neuron counting in the hippocampal CA1 area was performed at high magnification and analyzed by one-way ANOVA. The results showed that the TBI group had significantly fewer than the Sham group and the TBI + tFUS groups. (*P* < 0.01) (Fig. 6).

### VEGF

**Fig. 7:**
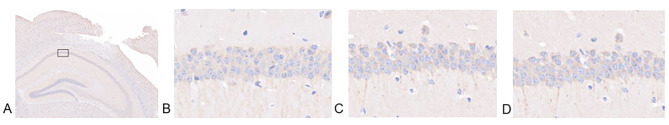
**A**. The protein expression of VEGF in the hippocampus of mice. **B**. the Sham group. **C**. the TBI + tFUS group. **D**. the TBI group

**Fig. 8:**
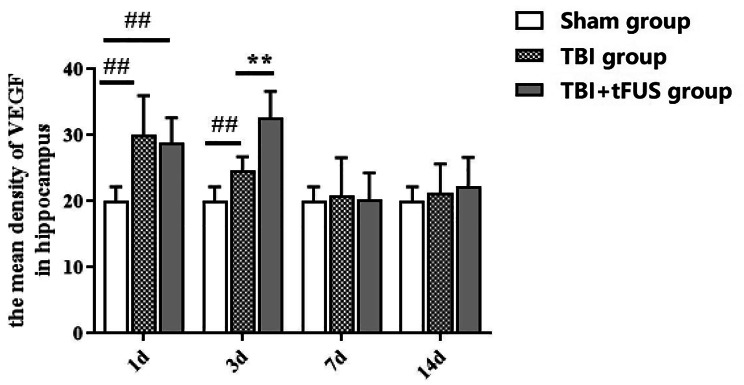
Results of VEGF detection in the hippocampus of Sham, TBI and TBI + tFUS groups were measured at 1d, 3d, 7d and 14d. Data are presented as mean ± SD. ##*P* < 0.01, Sham vs. TBI. **P < 0.01, TBI vs. TBI + tFUS.

The results of VEGF analysis in the hippocampus showed that compared with the sham group, the expression of VEGF in the hippocampus increased at 1st and 3rd days of TBI, the highest expression at 1st day, and then continued to decrease. Compared with the TBI group, tFUS intervention with VEGF was statistically significant only at 3rd day. At 7th and 14th days, there was no significant difference in the expression of VEGF in the hippocampus of each group (Figs. 7 and 8).

## Discussion

TBI is a fatal and disabling neurological lesion caused by trauma. Nerve regeneration and recovery after TBI are closely related to neovascularization as well as the formation and remodeling of the injured site. Restoration of blood supply is the basis of nerve repair, and blood vessels in the brain may serve as potential therapeutic targets to improve motor and cognitive deficits after TBIs [25].

The tFUS has been widely studied and used in the treatment of TBIs in recent years. Researchers are increasingly focusing on the potential use of tFUS to induce vascular remodeling. Various studies have demonstrated the therapeutic effects of tFUS on secondary brain injury in different ways. Studies have shown that tFUS has been used to stimulate revascularization of the myocardium after acute myocardial infarction. In mouse models, ultrasound combined with microbubble technology can increase micro vessel density and temporarily upregulate VEGF-A and insulin-like growth factor-1 in the myocardium, improving left ventricular function [26]. In a pig chronic myocardial ischemia model, Kenichiro et al. found that tFUS treatment enhanced multiple angiogenesis pathways, effectively increased capillary density and local myocardial vessels, and normalized myocardial dysfunction caused by ischemia, without any adverse reactions [13]. tFUS, which delivers ultrasound waves at a low intensity, can induce the angiogenic potential of endothelial cells [27]. Kang et al. found that after a period of tFUS treatment,human adipose derived stem cells/human umbilical vein endothelial cells that were co-cultured showed cell proliferation, assessed using the cell counting kit-8 assay, which together with the in vitro as well as in vivo immunofluorescence data on histological and immunohistochemical collagen and collagen/hydroxyapatite scaffolds, indicated that tFUS treatment has the ability to promote angiogenesis and also has therapeutic potential [9]. Wei-shen Su et al. [28] found that VEGF protein level in the LIPUS treatment group on day 4 was significantly increased by 60% compared with day 1. In the central nervous system, transient increases in vascular density and endothelial cell density of neovascularization were observed in the hippocampal tissues of rats treated with tFUS [29]. These findings suggest that tFUS is an effective, noninvasive treatment that stimulates angiogenesis and increases blood flow. Studies have also shown that tFUS was applied to a 25-year-old traumatic brain injury patient, and after 10 days of treatment, the patient’s level of consciousness gradually recovered [30].

In this experiment, we chose to use a controlled cerebral cortex strike, which had a precise, stable, and reliable strike intensity and can set the strike parameters by itself. The high-speed striking head impinges on the subdural cerebral cortex and causes injury. In our study, we used the severity of the damage induced by the parameters defined in the CCI mouse model: the damage induced by blow parameters at a speed of 3.5 m/s and a depth of 1 mm caused moderate TBI in the mice [31]. The use of this parameter avoided massive bleeding on the surface of the brain, reduced subdural hemorrhage, and created a suitable laser speckle image to damp down the surrounding area.

LSCI technology is a type of real-time laser reflection imaging of the intravascular red blood cells. It is a rapid, comprehensive and economic way of imaging, and this type of relatively simple imaging method, can provide a two-dimensional perfusion figure on the surface of the large area. The establishment of a mathematical model of the amount of blood flow perfusion can enable the use of the LSCI in clinical practice and the assessment of the blood supply in a wide range of tissues [12, 32].

Our results showed that tFUS treatment began to produce positive effects on CBF on day 1 after the CCI. In this study, LSCI was used to detect the blood supply in the injured cortex and contralateral cortex of the CCI mice, and the CBF in these two areas was measured to calculate the relative CBF. On day 7 after the CCI, the relative CBF in TBI group decreased to 38%, the lowest within 14 d. On day 7, the relative CBF in TBI + tFUS group remained near 80%. On the 14th day after CCI, the CBF in TBI group showed a slight spontaneous recovery. The results of the Nissl staining showed that the morphological structure of TBI + tFUS group was better, and the number of intact neurons was higher, which was consistent with the findings of other studies. Liu et al. found that spontaneous microvascular repair occurred in mice after CCI in the chronic stage and that the relative CBF in TBI + tFUS group was approximately 100% on day 14 [12]. From these results, we speculated that daily treatment with tFUS after traumatic brain injuries may further increase cerebral cortical blood perfusion, and that these increased blood perfusion results were consistent with better neurological recovery in mice. The NSS scores showed that although the mice in TBI group had spontaneous recoveries of their neurological functions after injuries, the recovery was not as good as that observed in TBI + tFUS group.

Our experimental results showed that on the first day after a TBI, the VEGF in the brain tissues around the injured area showed a significant increase, and it reached its peak in TBI + tFUS group on the third day after a TBI. These data suggest that the use of tFUS may promote angiogenesis and nerve repair by enhancing the expression and release of pro-angiogenic factors. Previous experimental studies have supported this hypothesis. Previous studies have also shown that cerebrovascular neovascularization is one of the most important and key mechanisms for functional recovery after a TBI [33]. A large amount of evidence has shown that in the process of angiogenesis, VEGF, an important angiogenic factor, plays an irreplaceable role. Many studies have found that VEGF can promote the proliferation, migration, and chemotaxis of vascular endothelial cells in various tissues and organs. Kenichiro et al. [13] proved for the first time that with the use of tFUS treatment there was a significant upregulation of VEGF mRNA expression, promotion of angiogenesis, and improvement in left ventricular myocardial ischemia. The histological results of Sharon et al. (Barzelai Sharon et al. et al. 2006) also showed that the number of blood vessels and proliferating cells in the moderate and severe ischemic groups treated with tFUS increased significantly. The VEGF expression also increased significantly during moderate ischemia, suggesting that tFUS may induce angiogenesis and vascular remodeling by upregulating the VEGF in injured peripheral brain tissues.

In conclusion, the experimental results indicate that tFUS treatment can promote cerebral vascular remodeling in the brain injury area, increase CBF, and reduce the secondary brain injury caused by hypoxia and ischemia, and especially, upregulate the expression of endothelium-derived angiogenic factors. Thus, tFUS can promote nerve repair and ultimately improve neurological outcomes after brain trauma.

This study had some limitations. The sample size and observation times of this experiment were relatively small, which may have caused some bias in the experimental results. Further research needs to be conducted by increasing the sample sizes and observation times in the future. The results of this study provide experimental evidence for our hypothesis, suggesting that tFUS may be an effective treatment method to prevent vascular failure after a TBI and to lay a solid theoretical foundation for the clinical use of tFUS in TBI.

## Data Availability

Data available on request from the authors.
